# Machine learning-based optimization of dual subthalamic nucleus and substantia nigra targeting in deep brain stimulation

**DOI:** 10.1038/s41531-026-01406-8

**Published:** 2026-05-25

**Authors:** Dallas Leavitt, Farzin Negahbani, Alireza Gharabaghi

**Affiliations:** 1 Institute for Neuromodulation and Neurotechnology (INN), University Hospital and University of Tübingen, Tübingen, Germany; 2 Max Planck-University of Toronto Center for Neural Science & Technology (MPUTC), Toronto, ON Canada; 3 Center for Digital Health (CDH), Tübingen, Germany; 4 Cognitive Science Center (CSC), Tübingen, Germany; 5 Center for Bionic Intelligence Tübingen Stuttgart (BITS), Tübingen, Germany; 6 German Center for Mental Health (DZPG), Tübingen, Germany

**Keywords:** Parkinson's disease, Movement disorders, Neurodegenerative diseases, Parkinson's disease

## Abstract

Advances in deep brain stimulation lead technology have created new opportunities for multi-site network modulation, including applications for freezing of gait, but systematic strategies for trajectory planning are lacking. We evaluated trajectories targeting the subthalamic nucleus (STN) and the simultaneous engament of the substantia nigra (SN), specifically its pars reticulata (SNr), which is considered as a potential target in Parkinson’s disease. By analyzing 612 electrode trajectories implanted with standard protocols, we found that 61% of trajectories engaged the SNr; simulating larger array spans or deeper implantation depth increased SNr engagement to 76%. We then trained Gaussian Process Classifiers to predict successful SNr engagement. Targeting a point ≥1.5 mm lateral to the medial STN border along Bejjani’s line, with an AC-PC angle ≥55° was associated with a ≥ 95% probability of yielding an SNr trajectory. This framework demonstrates that machine learning-assisted data analysis can generate planning principles for precise dual-site stimulation approaches.

## Introduction

Some neurological and psychiatric conditions may benefit from simultaneous deep brain stimulation (DBS) at multiple targets per hemisphere^1–4^. The availability of DBS leads capable of independent stimulation at multiple contacts, with larger inter-contact spacing or additional contacts^5^ has made it technologically feasible to refine targeting multiple brain loci with a single lead^6–9^. This capability has the potential to broaden the range of achievable stimulation effects while minimizing risk and cost. However, this advancement presents a new challenge for therapeutic planning: how can a single lead trajectory be optimized to reach multiple therapeutic targets?

An emerging application of multi-target DBS is dual targeting of the subthalamic nucleus (STN) and substantia nigra (SN), given the SN’s role in gait and balance^10^. Adjunct stimulation of the SN, in addition to the STN, has shown promise as a treatment for freezing of gait in Parkinson’s disease (PD), but results are mixed^9,11–17^.

Currently, dual STN-SN targeting is typically achieved by optimizing the trajectory for the STN alone, with the expectation that ventral contacts will incidentally reach the SN. Postoperative imaging, however, reveals that this approach is not consistently reliable in positioning contacts within the SN. Furthermore, existing approaches do not differentiate between the SN pars reticulata (SNr) and pars compacta (SNc), despite their distinct histological characteristics, connectivity patterns, and functional profiles^18–21^. Intra-SN heterogeneity, combined with variability in SN contact placement, may contribute to the inconsistent results in the STN-SN DBS literature. As dual STN-SN DBS becomes more widely adopted^22–24^, it is likely that distinct stimulation sweet spots will be identified within the SN^25^, necessitating refined approaches to therapeutic planning^26,27^.

While dual STN-SN targeting in current clinical practice may rely on direct visualization and electrophysiological signals, standard neuroimaging does not readily delineate the SNr-SNc boundary without the use of specialized sequences such as neuromelanin-sensitive MRI^28^. Similarly, although intraoperative microelectrode recordings can assist with lead placement^29^, their ability to reliably identify SN subregions may be limited in the pathological PD state^19,30^.

Machine learning (ML) offers an opportunity to address the complexities of therapeutic targeting in DBS^31–33^ by identifying trajectories that achieve the desired pattern of target engagement in a statistically principled manner. Rather than relying solely on iterative manual adjustments and experience-based heuristics, ML algorithms can analyze large datasets to uncover patterns that predict successful target engagement. This is particularly relevant because even for standard STN targeting, experts do not fully agree on a single optimal target location within the nucleus, with differences in preferred anterior-posterior position relative to Bejjani’s line and medial-lateral placement within the STN^34^. As a result, simply reporting the average coordinates or angles of successful trajectories would yield only one mean solution and would not capture the range of acceptable target choices used in clinical practice. Furthermore, simply taking the average of successful trajectories as our rule, without ML or some other analysis, does not tell us what success rate we can expect from that rule. In this context, ML has the potential to systematically inform trajectory planning and derive practical strategies for multi-target engagement, supporting the development of standardized heuristics across centers while preserving clinical flexibility. Importantly, such an approach can define trajectory corridors rather than a single fixed trajectory, allowing adjustment of implantation angles to maximize the probability of engaging a desired secondary target while preserving flexibility in the primary target selected by the expert.

In this study, we applied a Gaussian Process Classifier^35^ to a large dataset of reconstructed DBS leads of implanted PD patients to extract clinically applicable therapeutic planning principles for dual STN-SN targeting. By identifying reproducible imaging features, such as anatomical landmarks and trajectory angles, our goal was to translate complex spatial relationships into straightforward heuristics that can guide therapeutic planning without requiring real-time ML deployment. This approach uses ML as a tool to enhance, rather than replace, classical therapeutic planning.

We show that with larger interelectrode spacing or additional contacts, precise targeting of the SNr or SNc is achievable without compromising STN engagement. Furthermore, we propose practical rules of thumb for STN target and trajectory selection that enhance engagement of either the SNr or SNc, while remaining closely aligned with standard implantation procedures.

## Results

### Feasibility of dual STN-SN targeting

All measurements were performed in patient-native space to capture individual anatomical variability. For the primary analyses, a contact was considered to engage a region of interest if a 1 mm radius sphere centered on that contact contained any voxels from that region. To qualify as an SNr contact, the overlap with the SNr had to exceed the overlap with the SNc, and vice versa. A trajectory was considered to engage a region of interest if any of its contacts did so. Thus, while an individual contact could be classified as SNr or SNc, but not both, a trajectory could engage both regions.

**Fig. 1:**
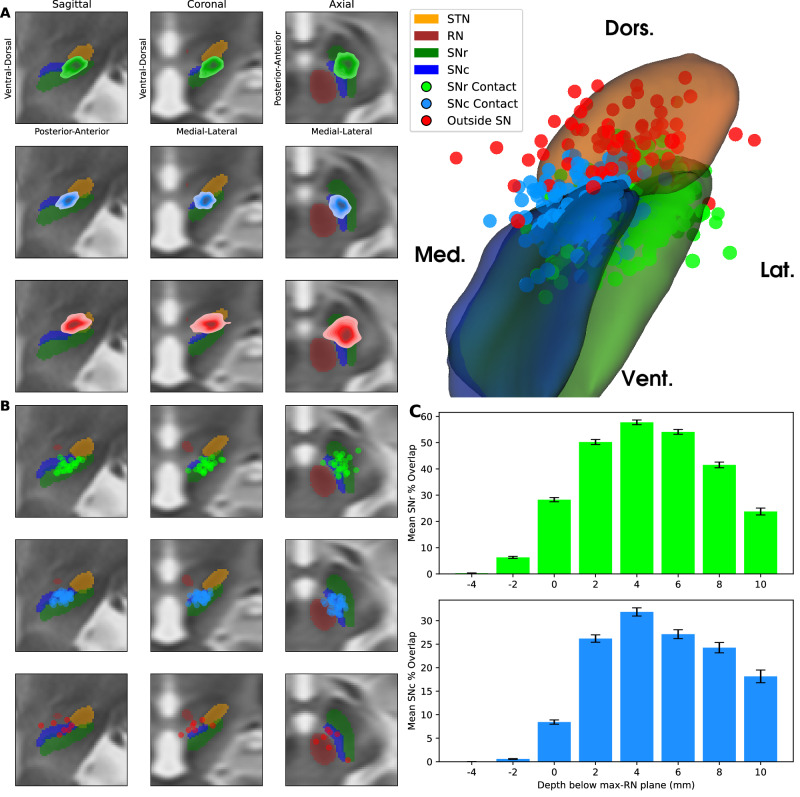
STN-targeted electrodes frequently reach the SN. **A** Kernel density estimates (KDEs) and scatter plots summarize the locations of the deepest contacts across trajectories, shown in sagittal, coronal, and axial slices (left) and on 3D models of the STN and SN (right). The STN is shown in orange, the red nucleus (RN) in brown, the SNr in green, and the SNc in blue. KDEs and contact locations are color-coded by classification: green, SNr contacts; blue, SNc contacts; red, contacts outside the SN. **B** For trajectories without an implanted contact in the SN, virtual contacts extending along the same trajectory are shown at the first position reaching the SN, or, if the SN was not reached, at the position closest to it. Colors are as in (**A**). **C** Mean percent overlap of the 1 mA volume of activated tissue (VAT) with the SNr (top, green) and SNc (bottom, blue) as a function of depth below the plane of maximal red nucleus cross-section (max-RN plane), measured along the trajectory. Each bin covers a depth range of 2 mm, centered on the value indicated by the horizontal axis. Error bars indicate SEM.

We first assessed whether a single trajectory could engage both the SN and STN during standard STN targeting. We attempted electrode reconstruction and brain normalization on 338 patients. A total of 306 patients passed our visual inspection quality checks, yielding 612 trajectories for analysis. Of these, 61% engaged the SNr and 36% engaged the SNc, with 10% engaging both nuclei, whereas 13% did not engage the SN at all (Fig. 1A).

We next simulated the effect of deeper implantation or extended electrode arrays by virtually extending each trajectory by 2 mm, 4 mm, and 6 mm, thereby generating three additional virtual contacts per lead (Fig. 1B). With virtual extension, SNr engagement increased to 76% and SNc engagement to 49%, with 27% of trajectories engaging both nuclei, while only 2% still failed to engage the SN.

To assess engagement quality beyond this binary classification, we simulated 1 mA volumes of activated tissue (VATs), approximated as spheres with a 2 mm radius, and measured percent overlap with the SNr and SNc as a function of contact depth. For both nuclei, overlap was greatest at a depth of 4 mm below the maximum red nucleus plane along the trajectory (Fig. 1C).

### Influence of electrode lead designs on SN engagement

We next examined the impact of implanted electrode design on the probability of SN engagement within the cohort. Compared with standard short-array span electrodes, defined here as leads with 0.5 mm intercontact spacing and four levels, implanted leads with larger array spans, due either to 1.5 mm spacing or additional levels, showed a higher success rate. SN contacts were present in 99% of trajectories with extended-array span electrodes, compared with 86% for short-array span electrodes. Virtual extension by 2 mm improved success rates for standard electrode designs, increasing SN engagement to 94%. The electrode models included in this study and their classification as standard or extended are listed in Table 1.

**Table 1:** Electrode models included in the dataset

Electrode model	*N* contact levels	Inter-level spacing (mm)	Exlectrode design
Boston Scientific Vercise Cartesia X	6	0.5	Extended
Boston Scientific Vercise Directed	4	0.5	Standard
Medtronic 3387	4	1.5	Extended
Medtronic 3389	4	0.5	Standard
Medtronic B33005	4	0.5	Standard
Medtronic B33015	4	1.5	Extended
Abbott/St. Jude Directed 6172	4	0.5	Standard
Abbott/St. Jude Directed 6173	4	1.5	Extended

Electrode models were classified as “extended“ if they had more than four contact levels or an inter-level spacing greater than 0.5 mm. All other models were classified as “standard“.

### STN engagement with dual-targeting trajectories

We then evaluated whether ventral SN contacts, including virtual ones, compromised the ability of more dorsal contacts to effectively target the STN. While optimal intra-STN targeting remains an open question, common goals include the motor compartment (MC)^35^ and, based on clinical experience at our center, a central target (CT) that overlaps reported sweet spots^26^.

**Fig. 2:**
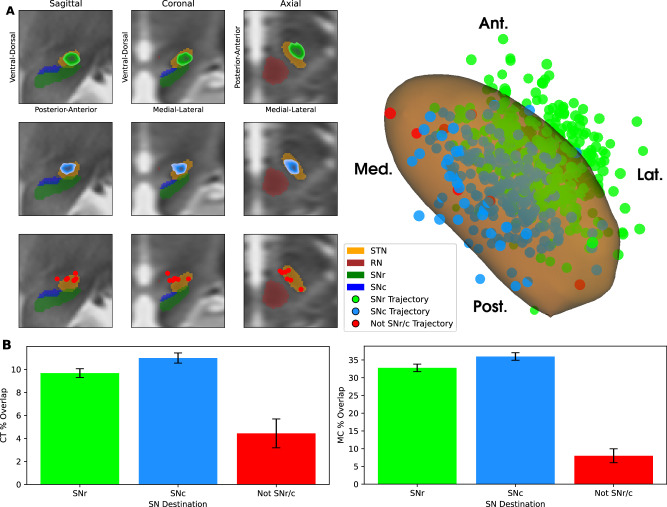
SN-engaging trajectories preserve effective STN targeting. **A** Kernel density estimates (KDEs) and scatter plots summarize the locations of the contacts with the greatest central target (CT) overlap for each trajectory, shown in sagittal, coronal, and axial slices (left) and on a 3D model of the STN (right). The STN is shown in orange, the red nucleus (RN) in brown, the SNr in green, and the SNc in blue. KDEs and contact locations are color-coded according to the trajectory classification: green, trajectories engaging the SNr; blue, trajectories engaging the SNc; red, trajectories not engaging the SN. **B** Mean percent overlap of the 1 mA volume of activated tissue (VAT) with the CT (left) and motor compartment (MC, right), stratified by nigral trajectory class. Error bars indicate SEM.

Among trajectories reaching the SNr, 79% also contained a contact in the MC and 49% in the CT (Fig. 2A). For SNc trajectories, these percentages were 88% and 59%, respectively. In contrast, trajectories not reaching the SN showed only 29% and 23% engagement of the MC and CT, respectively. These findings indicate that trajectories intersecting the SN were also generally well positioned within the STN.

VAT analysis supported this interpretation, showing greater overlap with the MC and CT for SN-reaching trajectories than for trajectories that did not engage the SN (Fig. 2B). Restricting the analysis to MC/CT trajectories, the rate of SNc engagement was 62% and the rate of SNr engagement was 74%, with 38% engaging both nuclei. Only 2% of MC/CT trajectories lacked any SN engagement.

### Trajectory characterization and targeting rule development

**Fig. 3:**
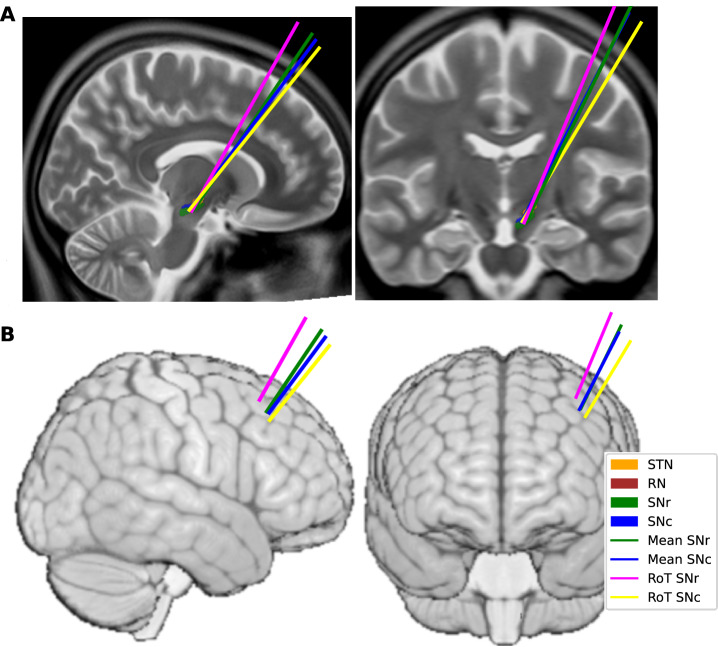
Mean and rule-of-thumb trajectories for SNr and SNc engagement. **A** Mean trajectories associated with SNr engagement (green) and SNc engagement (blue), together with the corresponding rule-of-thumb (RoT) trajectories derived from the classifier (magenta, SNr; yellow, SNc), shown on sagittal (left) and coronal (right) slices. **B** The same trajectories shown from the brain surface in lateral (left) and anterior (right) views.

To characterize trajectories passing through the SN, we measured midsagittal (MS) and anterior commissure-posterior commissure (AC-PC) angles. Trajectories engaging the SNr exhibited a mean (±SEM) MS angle of 22.3° (±0.23) and an AC-PC angle of 50.1° (±0.33). SNc trajectories had mean angles of 22.5° (±0.26) and 49.2° (±0.37), respectively (Fig. 3).

We also identified the axial slice at which the red nucleus appeared largest, the max-RN plane, and measured each trajectory’s intersection point relative to the intersection of Bejjani’s line with the medial STN border^36^. SNr trajectories had a mean (±SEM) intersection 2.0 mm (±0.07) lateral and 0.45 mm (±0.07) anterior to this reference point. SNc trajectories intersected 0.97 mm (±0.07) lateral and 0.66 mm (±0.07) posterior. Thus, the average SNr and SNc trajectories were distinct but remained spatially close to one another.

We then trained a Gaussian Process Classifier (GPC) to estimate the probability of SNr and SNc engagement based on patient-native features, namely the X and Y coordinates of the max-RN plane intersection and the MS and AC-PC angles. The SNr and SNc classifiers achieved 5-fold cross-validated accuracies of 90% and 87%, respectively. For trajectories for which the models were particularly confident, defined as a predicted class probability of at least 95%, 5-fold cross-validated accuracies increased to 99% and 98%, respectively.

**Fig. 4:**
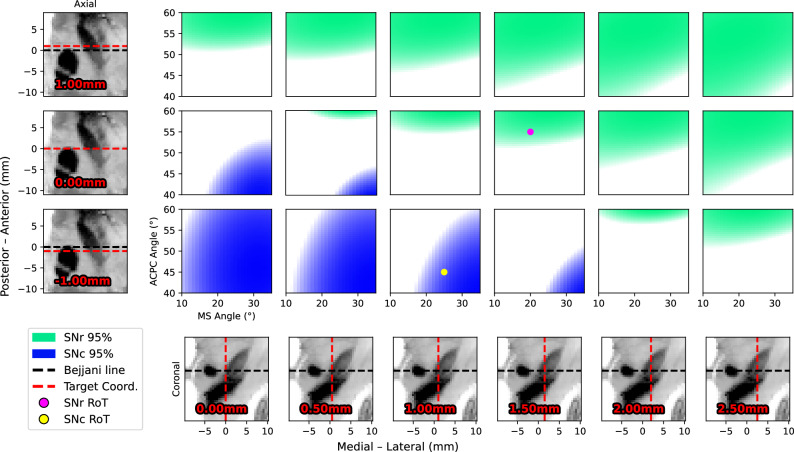
Gaussian Process Classifier-derived rules of thumb for STN-SNr and STN-SNc engagement. Predicted probabilities of SNr and SNc engagement are shown across a range of trajectories. Green and blue regions indicate combinations of midsagittal (MS) and anterior commissure-posterior commissure (AC-PC) angles associated with a predicted probability of at least 95% for SNr and SNc engagement, respectively, for the X and Y coordinates specified by column and row. The bottom row shows the X coordinate for each column on coronal slices, and the leftmost column shows the Y coordinate for each row on axial slices. Dashed red lines indicate the selected X and Y target coordinates used to define each panel. Bejjani’s line is indicated by a dashed black line. The magenta point marks a representative rule-of-thumb trajectory for SNr engagement, and the yellow point marks a representative rule-of-thumb trajectory for SNc engagement.

Using the trained models, we estimated class probabilities across a wide range of trajectories. For each combination of X and Y coordinates, we identified combinations of MS and AC-PC angles associated with a predicted probability of engagement of at least 95% (Fig. 4).

### STN-SNr targeting

We next derived simple rules of thumb that can be readily integrated into standard STN targeting workflows. To engage the SNr, one can select an STN target positioned at least 1.5 mm lateral to the medial STN border along Bejjani’s line at the max-RN plane. The corresponding trajectory should have an AC-PC angle of at least 55°. Additional rules, allowing for different medial-lateral and anterior-posterior target positions relative to Bejjani’s line or different trajectory angles, can be derived from the reference map shown in Fig. 4.

### STN-SNc targeting

To engage the SNc, an STN target located no more than 1.0 mm lateral and 1.0 mm posterior to the medial STN border along Bejjani’s line at the max-RN plane should be selected. The corresponding trajectory should have a midsagittal angle of at least 25° and an AC-PC angle of at most 45°. Further targeting adjustments can likewise be guided by the reference map shown in Fig. 4.

### Depth for STN-SN targeting

To derive a practical depth-based rule of thumb, we measured depth in patient-native space along the electrode trajectory, rather than along the z-axis, relative to the max-RN plane. Only trajectories that were anatomically on track to reach the SN at some point along their course were included, regardless of whether the actual implantation depth reached it, whereas off-target trajectories were excluded from this analysis.

We then trained a logistic regression model to classify the deepest contact as engaging the SN or not based solely on depth. We looked for the minimum depth at which the probability of engagement reached 95%. Complete placement of the lowest contact within the SN would require the lower border of that contact to lie 4.0 mm below the max-RN plane. This is in line with the depth-dependent SNr and SNc engagement profiles shown in Fig. 1C.

## Discussion

Our results show that dual targeting of the STN and SN with a single trajectory is feasible and can be optimized without compromising STN targeting. Specifically, optimizing implantation depth using electrode leads with larger interelectrode spacing or additional contacts allows engagement of the SN while preserving effective STN stimulation. Contrary to concerns that deep implantation may degrade STN targeting, we found that SN-reaching trajectories were actually more likely to optimally engage motor and central STN targets. These findings indicate that refined trajectories can preserve effective STN engagement while increasing the likelihood of nigral involvement. Clinical efficacy can be explored only in a limited way with retrospective data not specifically collected to evaluate the superiority of dual targeting, which is why this study was conceived as a methodological investigation rather than a clinical analysis.

Our findings underscore that an electrode exiting the ventral STN does not necessarily enter the SNr; a substantial proportion instead intersected the SNc. To enable more precise targeting, we derived and validated practical rules of thumb based on easily identifiable anatomical landmarks. These principles can be seamlessly integrated into standard STN planning protocols and demonstrate high accuracy in distinguishing SNr and SNc trajectories. When combined with large array-span electrodes, improved SNr targeting strategies offer the potential for more consistent and specific STN-SNr engagement. This, in turn, may enhance clinical outcomes of STN-SN DBS, building on promising results reported in prior studies^9,11–17^. While directional systems can compensate for minor deviations through steering, the intended SN target still needs to be reached anatomically. The present heuristics draw attention to the possibility of missing the SN even with deeper implantation and may help increase the likelihood of accurate SNr or SNc engagement. Although dual STN-SN stimulation is currently still a relatively uncommon application, we expect it to become more relevant as newer electrode designs with extended array spans make multisite targeting possible without additional implantation burden or clinical trade-offs. This may enable more systematic evaluation of combined STN-SN stimulation and help consolidate the clinical evidence for nigral stimulation, particularly when the intended target, such as the SNr, is reliably engaged. In this sense, the present work is intended to help pave the way toward more consistent and anatomically precise implementation of dual-target approaches.

It is important to note that the SNr and SNc are themselves heterogeneous structures with distinct connectivity and functional roles^36,37^. As demonstrated for the STN^38^, future research may reveal subregional functional zones within the SNr and SNc, necessitating even more refined targeting strategies. The framework and methodology presented in this study could be extended to support the development of such subregion-specific targeting approaches.

Machine learning contributed critically to our study. Rather than relying on predefined assumptions or manual inspection, our rules of thumb are the distillation of a targeting protocol learned directly from data. This approach allowed us to derive planning heuristics that are readily applicable in clinical practice without requiring access to the model. Importantly, machine learning served as an analytical tool to uncover reproducible patterns that may not be readily identifiable through subjective judgment or manual methods, such as drawing lines between anatomical landmarks in the STN and SN, a process that can be both time-consuming and prone to error. These heuristics complement rather than replace connectomic or sweet spot based approaches because they specify how to reach two predefined targets reliably with a single trajectory, regardless of whether those targets are defined anatomically, by sweet spot mapping, or through connectomic analyses. More broadly, the approach presented here is not restricted to STN-SN targeting. The same data-driven strategy could be extended to other emerging dual-target DBS applications, such as those proposed for essential tremor^39^ and epilepsy^40^, as well as to adaptive circuit targeting approaches, in which trajectory planning must account for engagement of multiple functionally relevant nodes along a single implantation path^41^.

While the GPC-based approach provided a robust framework for classification, several methodological considerations deserve attention. As a Bayesian method, GPCs revert to their prior, typically a 50/50 predicted class probability, in regions where training data is sparse. Consequently, if the classifier does not strongly “recommend” a particular trajectory, this may reflect limited representation of similar trajectories in the training set rather than true class ambiguity between SNr and SNc. This limitation primarily affects highly atypical trajectories, which are unlikely to occur in routine clinical practice.

Even after accounting for inter-patient variability, the determination of whether a trajectory intersects the SNr or SNc is influenced in part by the specific atlas used for SN segmentation^42^, though alternative atlases are available^43^. While all trajectory features were measured in patient-native space, segmentation of the SN and classification of trajectories as SNr or SNc still required normalization, which may introduce error. In the absence of a definitive ground truth, the accuracy of these segmentations is difficult to quantify within our dataset. The identified rules may not generalize to extreme anatomical variants. Incorporating patient-specific abnormalities through lesion-aware registration is a feasible but methodologically demanding next step in the future.

In summary, our study demonstrates that conventional STN trajectories often do not reach the intended SN subregion. However, dual STN-SN targeting with a single lead trajectory is technically feasible, can be systematically optimized, and does not compromise STN targeting accuracy. Machine learning enabled the identification of practical STN anatomical landmarks and straightforward trajectory planning principles for the reliable engagement of the SNr or SNc according to therapeutic goals. These findings lay the groundwork for the use of standardized and reproducible dual target DBS strategies in PD and related disorders, and may also inform future multisite and circuit-based DBS planning approaches beyond the specific STN-SN application studied here. Future prospective studies with clinical outcome validation will be essential to refine these approaches and establish their therapeutic relevance.

## Methods

This study was performed in accordance with the Declaration of Helsinki. Ethical approval (781/2015B02) was obtained from the University Hospital Tübingen Ethics Committee. Informed consent was waived, as all data were acquired during standard clinical care.

### Therapeutic procedure

The implantation site was identified by imaging-based direct targeting of the STN using preoperative MRI (T2-weighted and/or SWI)^44^. Patients were instructed to withhold antiparkinsonian medications overnight before surgery. Intraoperatively, electrophysiological recordings such as single-unit activity and/or local field potentials (LFPs) were used to confirm target localization. Postoperative CT imaging was performed for electrode reconstruction and confirmation of lead placement.

Single-unit recordings delineated STN entry and extent based on characteristic irregular or burst-firing patterns (20–60 Hz)^29^. The trajectory demonstrating the greatest STN span was selected for final implantation. When LFP guidance was used, placement was adjusted to position the two middle contacts within regions showing maximal beta-band (13–30 Hz) oscillatory activity, assessed through real-time intraoperative spectral analysis^45^. The final position of each lead was further verified with intraoperative clinical testing during stimulation, assessing therapeutic response and side-effect thresholds.

All surgeries included in this study were performed by the same neurosurgeon (A.G.), minimizing variability related to inter-surgeon differences in technique and decision-making. The data span a period of more than a decade, during which targeting strategies evolved in response to emerging evidence on optimal stimulation sites (e.g., sweet spots) and the increasing integration of connectomic information in DBS planning. Additionally, the dataset analyzed here included several different electrode types and designs (Table 1). While this variability introduces some heterogeneity, it also reflects real-world clinical practice and may enhance the generalizability of the findings. This study was conceived to investigate the methodological foundations of dual targeting rather than to make clinical claims, because retrospective datasets that are not designed to evaluate the superiority of dual targeting are inherently limited for addressing such questions.

### Lead and trajectory reconstruction

Lead reconstruction and VAT estimation were performed using Lead-DBS^46^. Preoperative MRI was co-registered to postoperative CT, followed by subcortical refinement. Patient native space images (T1/T2/FLAIR) were nonlinearly normalized to MNI ICBM 2009 NLIN ASYM space^47^ using SyN registration in Advanced Normalization Tools (ANTs)^48^. This process included two linear (rigid, affine) and two nonlinear SyN steps, the last (nonlinear) step targeting subcortical regions^49^. Although normalization was required for warping atlases into native space and for group visualization, all analyses were conducted in patient's native space rather than MNI space. DBS electrode artifacts were manually localized and corrected for brain shift by applying a refined affine transform between pre- and postoperative scans, restricted to subcortical regions, using the brain shift-correction module in Lead-DBS version 2.6^47^. Lead reconstruction and normalization quality was reviewed via visual inspection. In some cases, the quality of the reconstruction or normalization was poor and could not be ameliorated. These patients were not included in our final dataset.

The reconstructed ventralmost and dorsalmost contact locations defined the implantation trajectory norm (analogous to a 3D slope). This trajectory was extended virtually to simulate larger interelectrode spacing or additional contacts, enabling the generation of ‘virtual’ contacts. The trajectory vector also allowed estimation of cortical entry points and calculation of angles relative to the midsagittal and AC-PC planes. If the trajectory norm is a vector $l=\left(x,{y},{z}\right)$, where $x$ is in the right-left direction, $y$ is in the anterior-posterior direction, and $z$ is in the dorsal-ventral direction, and $n=\left(a,b,c\right)$ is the normal vector of the plane, then the angle between $l$ and ${n}$ is calculated as follows:

1 $${\rm{\theta }}={co}{s}^{-1}\left(\frac{l\cdot n}{\left|\left|l\right|\right|* \left|\left|n\right|\right|}\right)$$

where $\cdot$ denotes the vector dot product and ${||}.{||}$ denotes the Euclidean norm.

### Quantifying target engagement

Target regions were defined using published atlases. In the STN, the motor compartment (MC) was taken from ref. ^50^. An additional central target (CT) was constructed in the STN by identifying voxels within 0.75 mm of the MC-associative boundary and excluding the most ventral 50% of these voxels, consistent with DBS sweet spots reported in the literature^26^. In the SN, the SNr and SNc compartments were defined according to a published atlas^42^. All analyses were performed in patient-native space. A contact was considered to engage a target if a 1 mm radius sphere centered on that contact intersected the target. In cases of overlap with both SNr and SNc, the contact was assigned to the compartment with greater overlap. Thus, an individual contact could be classified as SNr or SNc, but not both, whereas a trajectory could engage both regions if different contacts intersected each compartment.

To complement this binary classification, we also quantified target engagement using modeled volumes of activated tissue (VATs). VATs for 1 mA stimulation were approximated as spheres with a 2 mm radius using established equations^50^ as implemented in Lead-DBS. VAT overlap was reported as the percentage of total VAT volume overlapping the target and served as a graded measure of engagement quality.

### Development of targeting rules for SNr and SNc

We sought to derive actionable targeting heuristics to differentiate SNr versus SNc trajectories. All trajectory angles and intersection coordinates used for model training were extracted in patient's native space, ensuring that the resulting targeting heuristics reflect individual anatomy. A Gaussian Process Classifier (GPC) was trained to classify SNr versus SNc trajectories based on four features:

- X coordinate in the axial plane at the maximum red nucleus diameter (max-RN)
- Y coordinate at the max-RN plane
- Angle to the midsagittal (MS) plane
- Angle to the AC-PC plane

Note that the origin of the X and Y coordinates (0, 0) was defined as the point where Bejjani’s line intersects with the medial border of the STN. This reference point was defined individually for each patient using native-space images.

Classifier performance was evaluated using 5-fold cross-validated accuracy. To derive practical high-confidence rules of thumb, we then focused on trajectory configurations for which the model predicted a probability of target engagement of at least 95%. The trained model was used to estimate class probabilities across a wide range of trajectories, enabling identification of acceptable combinations of MS angle and AC-PC angle for a given X and Y coordinate at the max-RN plane. Precision for these bounds was defined as the proportion of trajectories meeting the ≥95% probability threshold that correctly engaged the intended SN subregion.

We conducted a separate analysis to determine the required depth along the trajectory below the max-RN plane. For each patient with at least one contact (including virtual) located in the SN, the depth of contact 0 was calculated in native space. Each contact was then labeled as either ‘in SN’ or ‘not in SN’ based on the previously defined criterion. A logistic regression model was trained to classify contacts based on depth. We then used the model to identify the minimum depth at which the predicted probability of being in the SN reached ≥95%.

All analyses and visualizations were performed using custom Python scripts and 3D Slicer software^51^. The GPC was implemented using the GPy package^52^, based on the expectation propagation algorithm described by Williams and Rasmussen^35^. Logistic regression was done using the scikit-learn package^53^.

## Data Availability

Data and code supporting the key findings of this study will be made available by the first author to researchers upon request following publication, in accordance with ethical and legal regulations.
